# Comparative Analysis of Phylogenetic Relationships and Virulence Factor Characteristics between Extended-Spectrum β-Lactamase-Producing Escherichia coli Isolates Derived from Clinical Sites and Chicken Farms

**DOI:** 10.1128/spectrum.02557-22

**Published:** 2022-11-14

**Authors:** Chao Li, Xuan Chen, Zijing Ju, Cui Li, Ying Xu, Jiawei Ding, Yuting Wang, Peng Ma, Kui Gu, Changwei Lei, Yizhi Tang, Hongning Wang

**Affiliations:** a Animal Disease Prevention and Food Safety Key Laboratory of Sichuan Province, College of Life Sciences, Sichuan University, Chengdu, Sichuan, China; b Key Laboratory of Bio-Resource and Eco-Environment of Ministry of Education, College of Life Sciences, Sichuan University, Chengdu, Sichuan, China; c The First Affiliated Hospital of Chengdu Medical College, Chengdu, Sichuan, China; d Clinical Laboratory Department, Yan’an Hospital Affiliated with Kunming Medical University, Kunming, Yunnan, China; University of Guelph

**Keywords:** ESBL-*E. coli*, *bla*_CTX-M_, virulence factor, chicken farm, hospital

## Abstract

Antimicrobial resistance in bacteria is the most urgent global threat to public health, with extended-spectrum β-lactamase-producing Escherichia coli (ESBL-E. coli) being one of the most documented examples. Nonetheless, the ESBL-E. coli transmission relationship among clinical sites and chicken farms remains unclear. Here, 408 ESBL-E. coli strains were isolated from hospitals and chicken farms in Sichuan Province and Yunnan Province in 2021. We detected *bla*_CTX-M_ genes in 337 (82.62%) ESBL-E. coli strains. Although the isolation rate, prevalent sequence type (ST) subtypes, and *bla*_CTX-M_ gene subtypes of ESBL-E. coli varied based on regions and sources, a few strains of CTX-ESBL-E. coli derived from clinical sites and chicken farms in Sichuan Province displayed high genetic similarity. This indicates a risk of ESBL-E. coli transmission from chickens to humans. Moreover, we found that the high-risk clonal strains ST131 and ST1193 primarily carried *bla*_CTX-M-27_. This indicates that drug-resistant E. coli from animal and human sources should be monitored. As well, the overuse of β-lactam antibiotics should be avoided in poultry farms to ensure public health and build an effective regulatory mechanism of “farm to fork” under a One Health perspective.

**IMPORTANCE** Bacterial drug resistance has become one of the most significant threats to human health worldwide, especially for extended-spectrum β-lactamase-producing E. coli (ESBL-E. coli). Timely and accurate epidemiological surveys can provide scientific guidance for the adoption of treatments in different regions and also reduce the formation of drug-resistant bacteria. Our study showed that the subtypes of ESBL-E. coli strains prevalent in different provinces are somewhat different, so it is necessary to individualize treatment regimens in different regions, and it is especially important to limit and reduce antibiotic use in poultry farming since chicken-derived ESBL-E. coli serves as an important reservoir of drug resistance genes and has the potential to spread to humans, thus posing a threat to human health. The use of antibiotics in poultry farming should be particularly limited and reduced.

## INTRODUCTION

Bacterial infectious diseases have presented a significant challenge to clinical treatment and animal breeding practices, with Escherichia coli infections being the most prevalent (1). A 2019 report on the global survey of bacterial resistance indicated that E. coli is the leading cause of mortalities as a result of bacterial resistance (2). Bacterial resistance is a significant global challenge facing humans and animals; further, it is one of the most important clinical issues in world public health. Based on its strategy against antibiotic resistance, the World Health Organization (WHO) has selected seven antibiotic-resistant priority pathogens, including third-generation-cephalosporin (3GC)-resistant E. coli, which produces extended-spectrum β-lactamase (ESBL) (3). ESBL-producing E. coli (ESBL-E. coli) is commonly isolated from animals and humans and even in environments with little or no human activity (4, 5). Further, the overuse of antibiotics exacerbates the development of ESBL-E. coli strains, which often result in antibiotic treatment failure and limit clinical and animal drug use. Studies have shown that ESBL-E. coli is resistant to penicillin, aminopenicillin, and cephalosporins, including the approved veterinary drugs 3GC ceftiofur and cephalosporin and the fourth-generation cephalosporins (6). Humans and animals are closely related based on the One Health perspective. Long-term use of antibiotics in animals causes the development of bacterial resistance in animals, and in turn, these resistant bacteria may directly or indirectly transmit drug resistance genes to humans. This suggests that improving animal health promotes human health.

Several studies indicate that chickens are an important reservoir of ESBL genes, attracting significant global concern. ESBL-E. coli causes infection in broilers and layers, which is a critical contaminant in retail chicken meat. As a principal major source of protein, chicken is one of the largest global meat sources, with extensive consumption (7). The poultry farming industry is one of the fastest-growing industries across the globe. Many studies have identified antibiotic residues in poultry, potentially contributing to the development of resistance in human pathogens (8). Under the “farm to fork” strategy, antibiotic use in animals has been banned or reduced globally; this has resulted in a reduced prevalence rate of ESBL-E. coli derived from animals (9–11). Nonetheless, antibiotics are still the predominant therapeutic intervention for bacterial infectious diseases. Reports indicate that the overall use of antibiotics will gradually increase in the next decade, and 73.7% of them will be used in livestock (12, 13). The extensive use of antibiotics in animal farming has increased the proliferation of drug-resistant bacteria, which can be transmitted to humans via the food chain, water, or air.

The prevalence of β-lactam resistance derived from chicken farms significantly varies between countries and regions. For instance, the β-lactam resistance rate of E. coli derived from chickens in northern India is 49% (14), whereas the cefotaxime resistance rate of E. coli derived from chickens in Bangladesh is 78.1% (15). Also, studies have shown that the prevalence characteristics of β-lactam resistance vary in different specific regions of a country. For example, the cefotaxime resistance rate of E. coli isolated from chicken farms in different regions of South Korea ranged between 17.5% and 51.4% (16). Notably, *bla*_CTX-M_ is the most prevalent β-lactam resistance gene in ESBL-E. coli (17), and CTX-ESBL-E. coli has been isolated from humans and animals across the globe (18). So far, over 200 subtypes of *bla*_CTX-M_ have been identified, and the prevalent subtypes vary in different regions and sources. For instance, *bla*_CTX-M-1_ is the dominant resistance gene in ESBL-E. coli isolated from cattle in Germany, whereas *bla*_CTX-M-15_ is the dominant resistance gene in cattle in England (19). Moreover, studies indicate that *bla*_CTX-M-1_ is the dominant resistance gene in ESBL-E. coli isolated from farmed animals in Europe, whereas *bla*_CTX-M-15_ is predominant in the United States (20, 21). The *bla*_CTX-M-14_ gene is less prevalent in Europe; however, it is prevalent in poultry, animals, and humans in Asia (22). As such, the epidemiological investigation of *bla*_CTX-M_ is complex.

The majority of E. coli strains are intestinal commensals in humans and animals. Nevertheless, some clonal strains with a high risk of pathogenicity have been discovered, which cause multiple infectious diseases and even death. The extraintestinal pathogenic E. coli (ExPEC) ST131 belongs to the high-risk clonal subgroups, which carry several virulence genes and cause severe extraintestinal infections, including bloodstream infections, urinary tract infections, pneumonia, and neonatal meningitis. Of note, *bla*_CTX-M-15_ is the dominant resistance gene in E. coli ST131, originally identified in Canada, France, Switzerland, Portugal, Spain, Kuwait, Lebanon, India, and South Korea (23, 24) and presently having a global distribution trend (25). Unlike the non-ST131 E. coli, E. coli ST131 has a larger number of ExPEC-associated virulence factor genes. Moreover, E. coli ST131 effectively colonizes the intestine, bladder, and kidney of humans and food animals, including chickens and wild birds (26–29). Since ST131 carries numerous virulence factor genes and β-lactam resistance genes, once it is widely distributed in poultry, it causes treatment failure and huge economic losses to the poultry farming industry, posing a significant threat to human health.

Although transmission of pathogenic bacteria from poultry to humans has been reported, whether bacterial resistance can spread from poultry to humans remains unclear (30). ESBL-E. coli is widespread, but whether there is a clonal transmission relationship between ESBL-E. coli isolates derived from clinical sites and chicken farms is unclear. Therefore, it is important to analyze the sources of ESBL-E. coli isolates and their drug resistance genes to explore the transmission relationship between ESBL-E. coli isolates derived from humans and chickens from the perspective of One Health. In this study, we isolated and analyzed the prevalence of ESBL-E. coli derived from clinical sites and chicken farms in Sichuan and Yunnan provinces of China to understand the differences in bacterial resistance between different sources and provide epidemiological evidence of β-lactam resistance. Therefore, our findings will provide recommendations for poultry breeding and medical clinical interventions, as well as mitigation strategies for antibiotic resistance and its effects.

## RESULTS

### Isolation of CTX-ESBL-E. coli.

**FIG 1:**
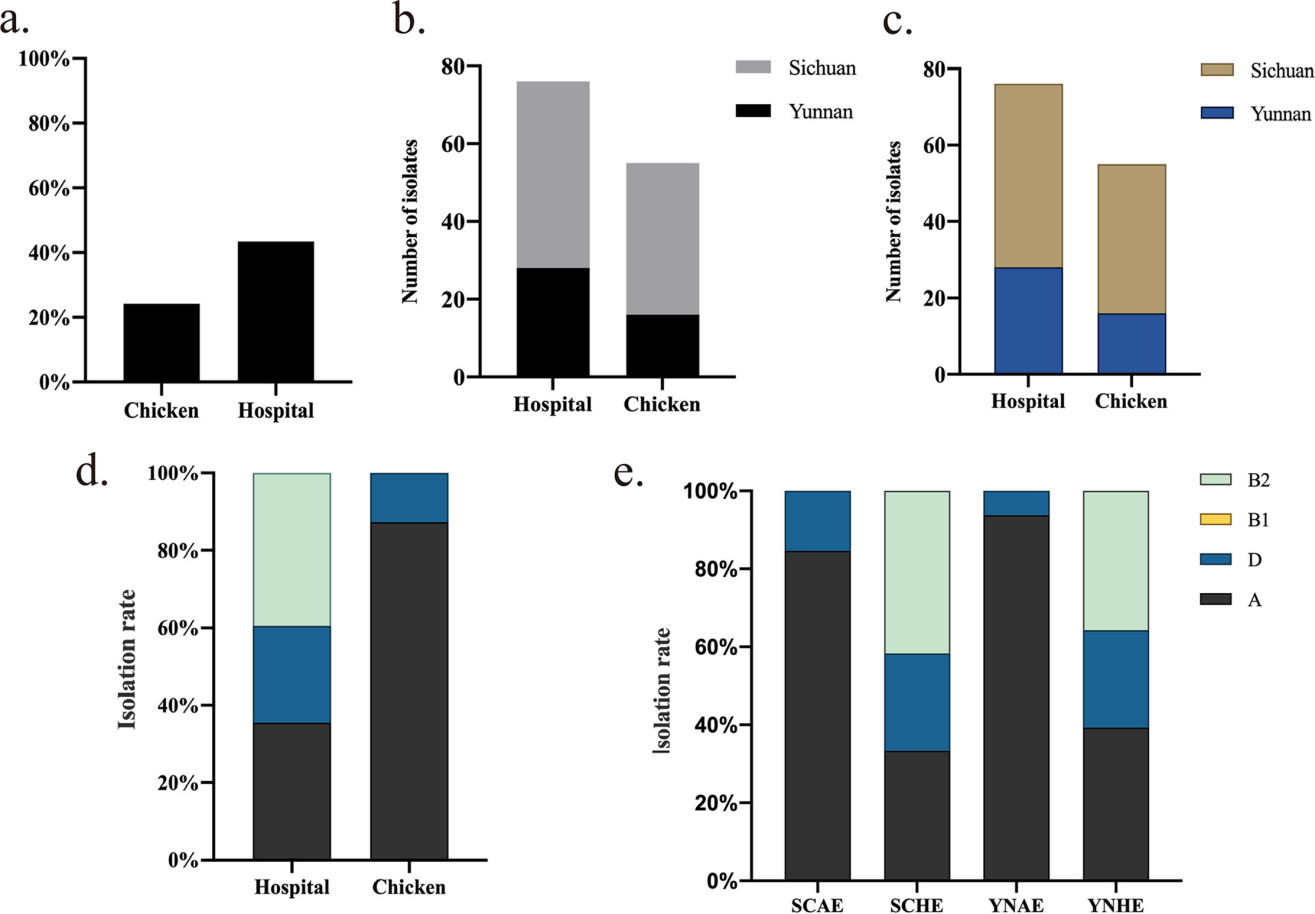
Isolation and phylogenetic analysis of CTX-ESBL-E. coli. (a) Isolation rate of CTX-ESBL-E. coli in chicken farm-derived isolates and clinical isolates. (b) Isolation rate of CTX-ESBL-E. coli in chicken farm-derived isolates and clinical isolates in Sichuan Province and Yunnan Province. SCAE and YNAE indicate the chicken farm-derived CTX-ESBL-E. coli in Sichuan Province and Yunnan Province, respectively, SCHE and YNHE indicate the clinical CTX-ESBL-E. coli in Sichuan Province and Yunnan Province, respectively. (c) Distribution of 131 CTX-ESBL-E. coli isolates used for whole-genome sequencing. (d and e) Isolation rates of four phylogenetic groups of 131 CTX-ESBL-E. coli isolates.

A total of 1,038 E. coli strains were isolated in Sichuan and Yunnan provinces from January to September 2021, i.e., 594 strains isolated from chicken farms (404 strains from Sichuan Province and 190 strains from Yunnan Province) and 444 clinical isolates (100 strains from Sichuan Province and 344 strains from Yunnan Province). Among these, 408 isolates were identified as ESBL-E. coli, accounting for 39.3% of the total isolates, including 204 isolates from chicken farms (151 strains from Sichuan Province and 53 strains from Yunnan Province) and 204 clinical isolates (48 strains from Sichuan Province and 156 strains from Yunnan Province). *bla*_CTX-M_ ESBL genes were detected, and 337 (82.62%) ESBL-E. coli isolates carried *bla*_CTX-M_ genes, including 144 isolates from chicken farms (107 strains from Sichuan Province and 37 strains from Yunnan Province) and 193 clinical isolates (48 strains from Sichuan Province and 145 strains from Yunnan Province). The detection rate of CTX-ESBL-E. coli in chicken farm-derived isolates was 24.24% and in clinical isolates was 43.47% (Fig. 1a). The clinical detection rate of CTX-ESBL-E. coli was higher in Sichuan Province (48%, 48/100) than in Yunnan Province (42.15%, 145/344). The detection rate of chicken farm-derived CTX-ESBL-E. coli in Sichuan Province (26.49%, 107/404) was also higher than that in Yunnan Province (19.47%, 37/190) (Fig. 1b). Finally, 131 CTX-ESBL-E. coli isolates (55 strains isolated from a chicken farm and 76 clinical isolates) with higher levels of resistance to cefotaxime (>128 μg/mL) were chosen (Fig. 1c) based on susceptibility testing results and used for whole-genome sequencing (WGS) (see Table S1 in the supplemental material).

### Determining the phylogenetic groups of E. coli strains.

Phylogenetic analyses indicated that E. coli strains fell into four primary phylogenetic groups (A, B1, B2, and D) with various pathogenicities. Strong pathogenicity was found in E. coli in the B2 and D groups, whereas weak pathogenicity was found in E. coli in the B1 group. The A group relates to animal and human gut commensals. Phylogroup analysis of the 131 CTX-ESBL-E. coli isolates discovered that 57.25% (48 CTX-ESBL-E. coli isolates from chicken farms and 27 clinical isolates) were classified in the A group, 19.85% (7 CTX-ESBL-E. coli isolates from chicken farms and 19 clinical isolates) were classified in the D group, and 22.9% (30 clinical isolates) were classified in the B2 group with the strongest pathogenicity (Fig. 1d). These results show that the clinically isolated CTX-ESBL-E. coli strains are more pathogenic than chicken farm-derived isolates. The isolation rate of pathogenic clinical CTX-ESBL-E. coli was higher in Sichuan Province (66.67%, 32/48) than in Yunnan Province (60.71%, 17/28). However, the isolation rate of chicken farm-derived CTX-ESBL-E. coli classified in the D group from Sichuan Province was 15.38% (6/39), which was significantly higher than that from Yunnan Province at 6.25% (1/16) (Fig. 1e). In addition, all 30 CTX-ESBL-E. coli strains in the B2 group were isolated from hospitals, illustrating the strong pathogenic properties of the clinically isolated CTX-ESBL-E. coli. Nevertheless, 20% of CTX-ESBL-E. coli isolates derived from chicken farms belong to the D group with specific pathogenicity. Avian-pathogenic E. coli was reported to transmit virulence factors to pathogenic E. coli outside the intestinal tract. Thus, continuous surveillance of pathogenic E. coli from different sources is the key to understanding potential transmission routes between animals and humans (31).

### SNP evolutionary analysis.

**FIG 2:**
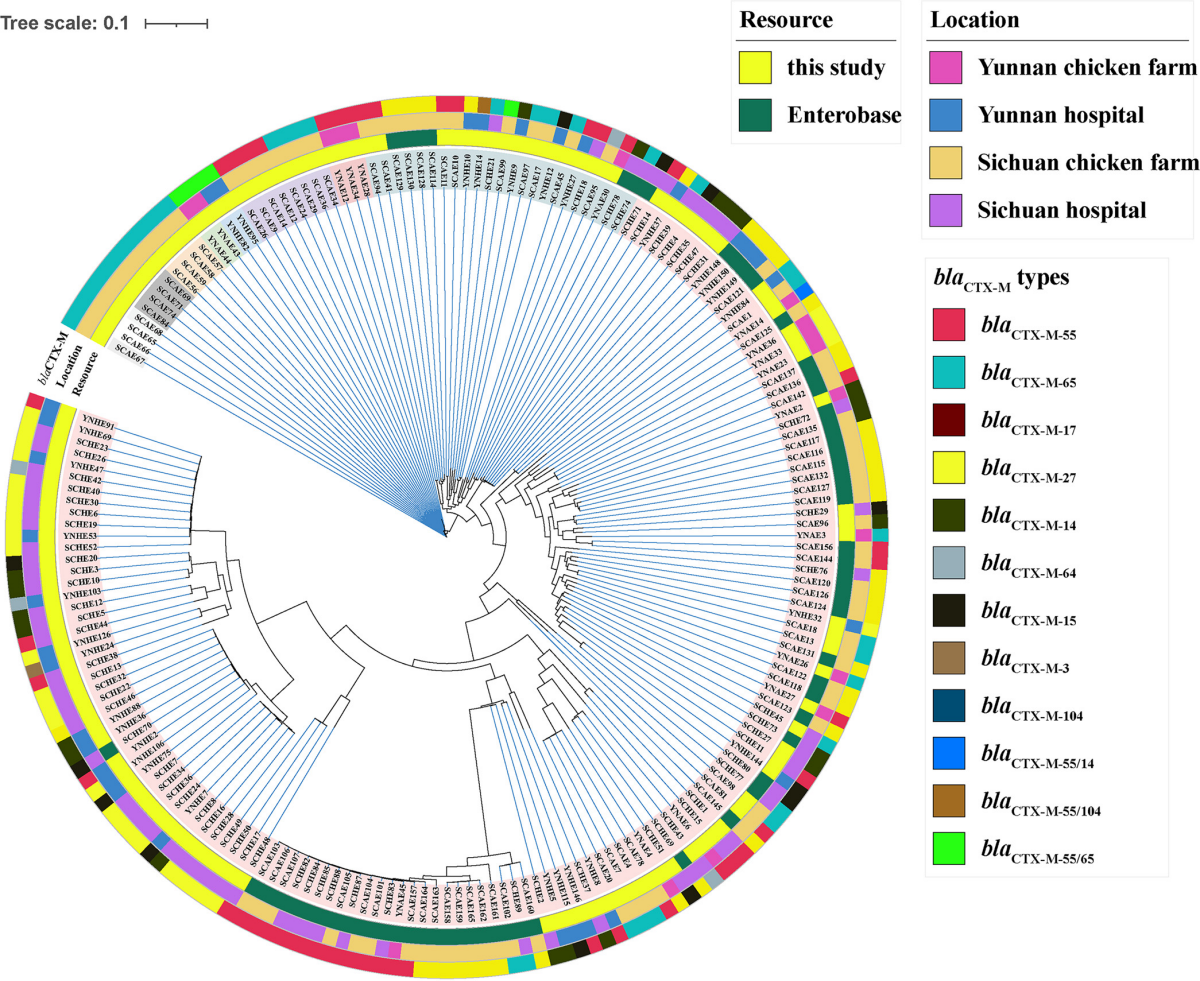
Single nucleotide polymorphism evolutionary analysis of CTX-ESBL-E. coli isolates. The SNP evolution tree was created with CGE CSI Phylogeny 1.4, which was observed using the Interactive Tree Of Life (iTOL v6, https://itol.embl.de/). The genome sequence of E. coli C600 (EC600, GenBank accession no. CP031214.1) was used as a reference.

A total of 63 CTX-ESBL-E. coli genome sequences from Yunnan and Sichuan provinces were downloaded from the EnteroBase database and were combined with 131 isolated CTX-ESBL-E. coli strains to construct a single nucleotide polymorphism (SNP) evolution tree (Fig. 2 and Table S2). SNP evolution analysis showed that the minimum number of SNPs was 0 and the maximum number of SNPs was 55,630; 87/131 strains had high similarity to strains in the EnteroBase database. A threshold of 5 SNPs among isolates was considered a clonal relationship, and the isolates were likely to have an epidemiological relationship. Figure 2 illustrates a higher similarity in the clinically isolated CTX-ESBL-E. coli strains from two provinces than in the CTX-ESBL-E. coli strains derived from chicken farms. Similarly, a higher similarity was also noted between CTX-ESBL-E. coli strains derived from chicken farms in two provinces; we also found strains of the same clone. Moreover, a few clinical and chicken farm-derived CTX-ESBL-E. coli isolates from Sichuan Province demonstrated genetic similarity (Table S2). Although significant SNP differences between CTX-ESBL-E. coli isolates from humans and chicken farms were observed, we noted a high genetic similarity in human and chicken farm-derived CTX-ESBL-E. coli isolates in the same region. This indicates that animal-human transmission of drug-resistant isolates easily occurs within the same region.

### MLST analyses.

**FIG 3:**
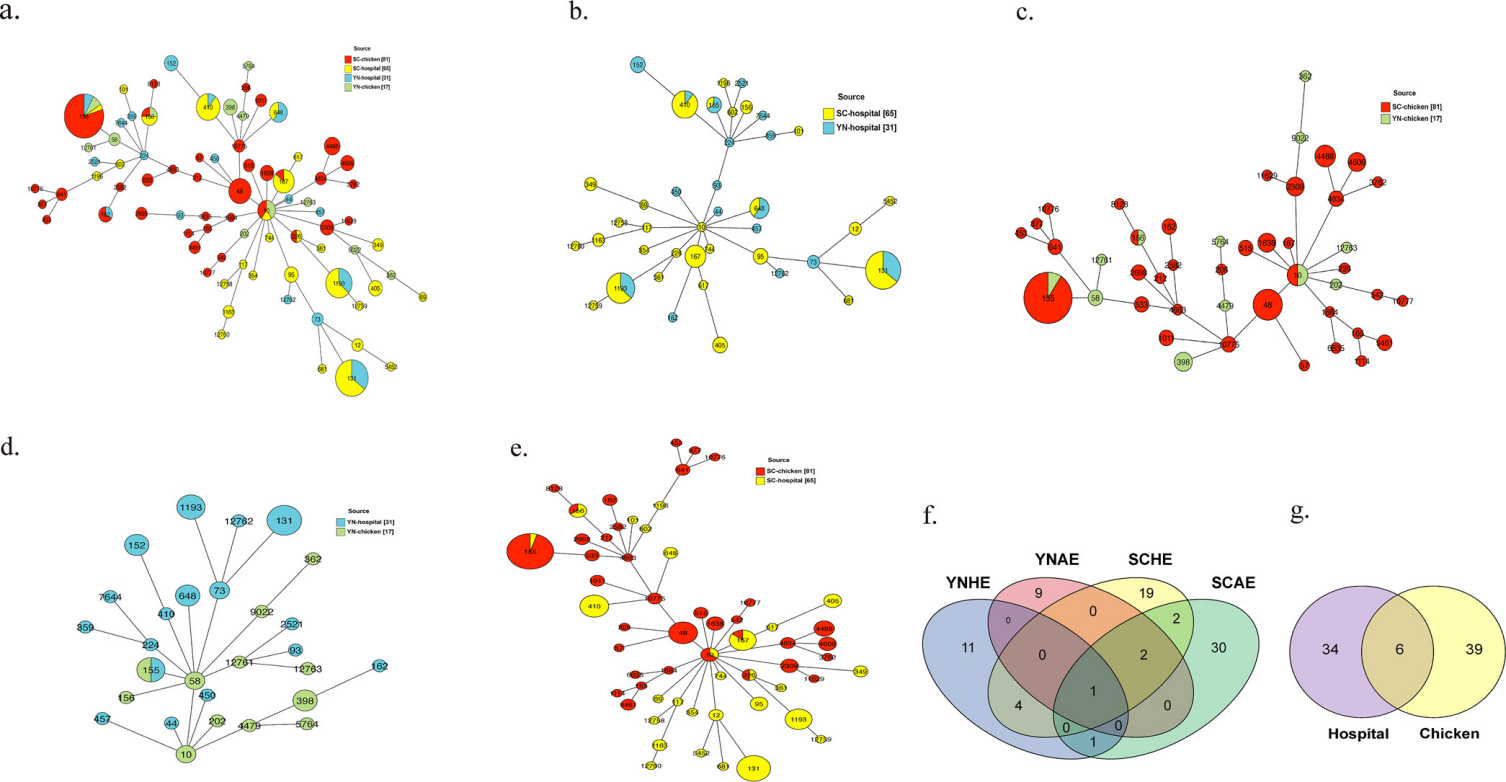
Minimum spanning tree analysis based on multilocus sequence typing analyses of CTX-ESBL-E. coli. (a) ST distribution of CTX-ESBL-E. coli isolates. (b to e) ST distribution of CTX-ESBL-E. coli isolates from different regions and sources. (f and g) Number of shared STs in the CTX-ESBL-E. coli isolates.

Multilocus sequence typing (MLST) analysis of 194 isolates (Table S3) was performed and revealed significant diversity among these isolates. Notably, 78 sequence types (STs) were shared in 194 CTX-ESBL-E. coli isolates (Fig. 3a). ST155 (25 strains), ST131 (18 strains), ST1193 (11 strains), ST410 (9 strains), and ST48 (7 strains) were the dominant STs. A total of 32 STs were categorized in clinically isolated ESBL*-* E. coli (Fig. 3b), with ST131, ST410, and ST1193 being the predominant STs. A total of 24 STs were classified in the chicken farm-derived CTX-ESBL-E. coli isolates (Fig. 3c), with ST155 and ST48 being the dominant STs. No other cross-STs were present in both clinical and chicken farm-derived CTX-ESBL-E. coli isolates in Yunnan Province, except ST155 (Fig. 3d), whereas ST156, ST155, ST10, ST167, and ST226 were present in both clinical and chicken farm-derived CTX-ESBL-E. coli isolates in Sichuan Province (Fig. 3e). The dominant ST of clinical CTX-ESBL-E. coli was ST131, followed by ST1193, whereas ST155 was the dominant ST of chicken farm-derived CTX-ESBL-E. coli. In contrast, only ST155 E. coli was distributed in four sources (Fig. 3f). Six STs (ST162, ST156, ST10, ST155, ST167, and ST226) in chicken farm- and clinical site-derived CTX-ESBL-E. coli isolates (Fig. 3g) were found, suggesting that these six STs of E. coli are likely to spread between humans and chicken farms at a higher risk. The ubiquitous presence of ST155 E. coli distributed in four sources indicates the sequence type’s transmission benefits. In total, ST155 was the predominant ST in two provinces, followed by ST131. ST1193 and ST410 were found only in clinical isolates, whereas ST48 was found only in chicken farm-derived CTX-ESBL-E. coli in Sichuan Province. The above findings illustrate that chicken farm-derived and clinical isolates of CTX-ESBL-E. coli exhibit a certain risk of cross-transmission, especially in the same area. Therefore, there is an urgent need for a comprehensive prevalence survey of CTX-ESBL-E. coli in the respective areas. Additionally, we discovered 6 novel STs (Tables S4 and S5), of which 2 were in chicken farm-derived CTX-ESBL-E. coli isolates (ST12761 and ST12763), whereas 4 were in clinical site-derived CTX-ESBL-E. coli isolates (ST12758, ST12759, ST12760, and ST12762).

### AST and antimicrobial resistance gene analysis.

**FIG 4:**
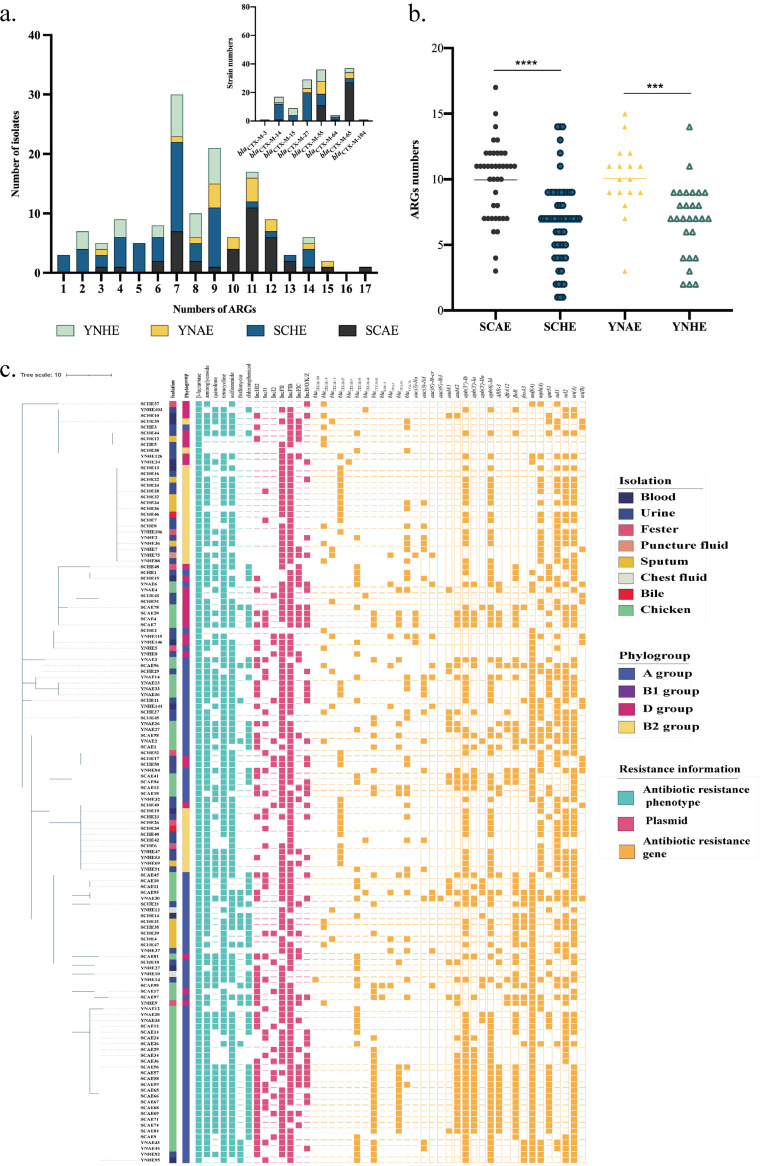
Antimicrobial sensitivity test, antimicrobial resistance genes, and plasmid replicon analysis of CTX-ESBL-E. coli. (a) Numbers of various drug resistance genes carried by CTX-ESBL-E. coli isolates from different regions and sources. The histogram in the upper right corner indicates the number of strains carrying different *bla*_CTX-M_ genes. ARG, antimicrobial resistance gene. (b) Comparative analysis of the number of drug resistance genes carried by CTX-ESBL-E. coli from different regions and sources. ***, *P* ≤ 0.001; ****, *P* ≤ 0.0001. (c) Antibiotic resistance phenotypes, antibiotic resistance genes, and plasmid replicon analysis of the CTX-ESBL-E. coli. The evolutionary tree was constructed using GrapeTree of the EnteroBase site based on the STs and visualized using iTOL v.6.

Ceftriaxone (CRO), gentamicin (GEN), levofloxacin (LVX), tetracycline (TET), sulfamethoxazole (SXT), fosfomycin (FOS), and florfenicol (FFC) were used to perform an antimicrobial sensitivity test (AST) based on CLSI experimental guidance. Consequently, 92.37% (12/131) of CTX-ESBL-E. coli isolates showed multiple drug resistance (Fig. 4; Table S5). Besides the resistance to β-lactam antibiotics, the resistance rates against tetracyclines, aminoglycosides, sulfonamides, chloramphenicol, and fosfomycin were 86.26% (113/131), 84.73% (111/113), 80.92% (106/131), 41.98% (55/131), and 14.5% (19/131), respectively. To understand the antimicrobial resistance characteristics of these strains, 131 strains of CTX-ESBL-E. coli were used for whole-genome sequencing. The antibiotic resistance genes and plasmids in the genome sequence were searched using ResFinder and PlasmidFinder on the Center for Genomic Epidemiology (CGE) website, and we analyzed 33 antibiotic resistance genes (including 12 β-lactam resistance genes, 10 aminoglycoside resistance genes, 1 rifampicin resistance gene, 1 diaminopyrimidine resistance gene, 1 chloramphenicol resistance gene, 1 fosfomycin resistance gene, 3 tetracycline resistance genes, 1 macrolide resistance gene, 1 quinolone resistance gene, and 2 sulfonamide resistance genes) and 7 plasmids (Fig. 4c). Further, 131 CTX-ESBL-E. coli isolates from different sources were classified and assessed for the number of various drug resistance genes (Fig. 4a): 30 strains had 7 drug resistance genes (including 8 chicken farm isolates and 22 clinical isolates) and 21 strains had 9 drug resistance genes (including 5 chicken farm isolates and 16 clinical isolates).

The plasmid is an important mobile genetic element, housing the majority of the drug resistance genes encoding ESBL. The common replicons include IncF, IncI, IncN, IncHI1, and IncHI2 (6). Most strains in this study carried IncFII and IncFIb plasmids, implying that the drug resistance genes carried by these strains have the potential for horizontal transfer. Moreover, we calculated the *bla*_CTX-M_ subtypes carried in 194 strains and eventually discovered 12 *bla*_CTX-M_ subtypes. The predominant subtypes include *bla*_CTX-M-14_, *bla*_CTX-M-27_, *bla*_CTX-M-55_, and *bla*_CTX-M-65_. A total of 37 E. coli strains had *bla*_CTX-M-65_, whereas 36 E. coli strains carried *bla*_CTX-M-55_. The clinical CTX-ESBL-E. coli isolates primarily carried *bla*_CTX-M-14_, *bla*_CTX-M-27_, and *bla*_CTX-M-55_, whereas the chicken farm-derived CTX-ESBL-E. coli isolates mainly carried *bla*_CTX-M-55_ and *bla*_CTX-M-65_. This shows the different prevalent subtypes of CTX-ESBL-E. coli from the two sources. Also, some strains carried two subtypes of *bla*_CTX-M_ in their genome sequences. A total of 7 strains carrying two subtypes of *bla*_CTX-M_ were identified, including Sichuan Province chicken farm-derived isolate SCAE99 (*bla*_CTX-M-65_, *bla*_CTX-M-55_) belonging to ST2582, Yunnan Province farm-derived isolate YNAE14 (*bla*_CTX-M-65_, *bla*_CTX-M-14_) belonging to ST202, YNAE43 and YNAE44 (*bla*_CTX-M-65_, *bla*_CTX-M-55_) belonging to ST155, Yunnan clinical isolates YNHE82 and YNHE95 (*bla*_CTX-M-65_, *bla*_CTX-M-55_) belonging to ST155, and YNHE14 (*bla*_CTX-M-104_, *bla*_CTX-M-55_) belonging to ST224. These findings show that β-lactam-resistant ST155 E. coli is extensively distributed and easily carries the *bla*_CTX-M_ gene, which is conducive to horizontal gene transfer. Subsequently, we analyzed the number of drug resistance genes in strains from different sources. We found that chicken farm-derived isolates contained significantly more drug resistance genes than clinical isolates in two provinces (Fig. 4b). The above results confirm that chicken farm-derived E. coli is an important drug resistance gene repository. Therefore, measures should be taken to promptly and effectively regulate the severe situation of bacterial drug resistance in chicken farms.

### Analysis of virulence-associated genes.

**FIG 5:**
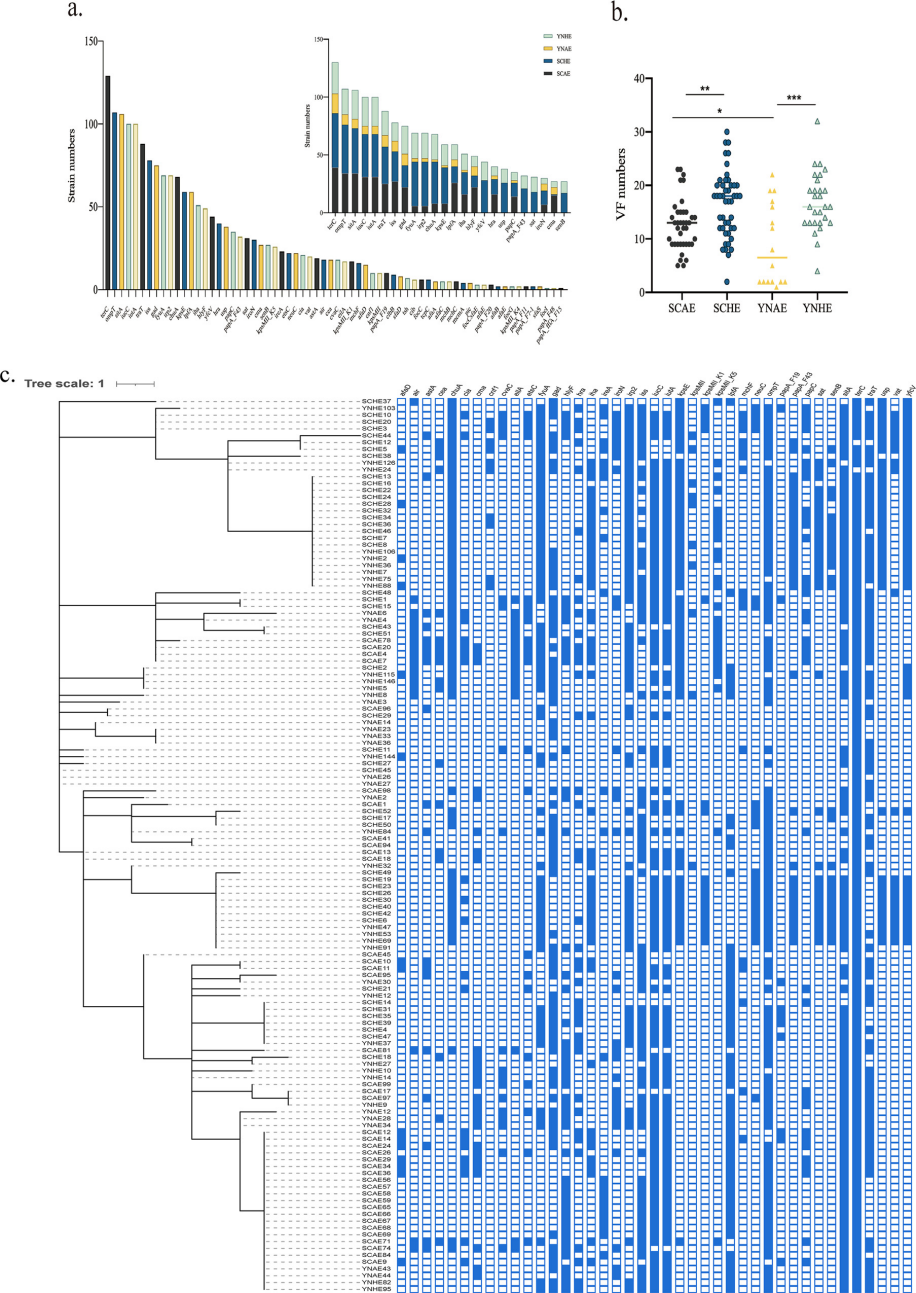
Analysis of virulence-associated genes. (a) Number of virulence-associated genes in CTX-ESBL-E. coli isolates from various sources. The histogram in the upper right corner indicates the number of the 24 virulence factors with the highest detection rate carried by the CTX-ESBL-E. coli isolates from different sources. (b) Comparative analysis of the number of virulence-associated genes carried by CTX-ESBL-E. coli isolates from different sources; *, *P* ≤ 0.05; **, *P* ≤ 0.01; ***, *P* ≤ 0.001. (c) Virulence-associated gene distribution in the CTX-ESBL-E. coli isolates, the evolutionary tree was constructed using GrapeTree of the EnteroBase site based on the STs and visualized using iTOL v.6.

The virulence factors were analyzed to understand the genomic characteristics of 131 CTX-ESBL-E. coli strains (Fig. 5 and Table S6). A total of 66 virulence factors (Fig. 5a) were identified in 131 E. coli strains using VirulenceFinder on the CGE website. *terC*, an anti-tellurium ion protein-coding gene, had the highest detection rate, followed by outer membrane protein T (*OmpT*) and *sitA* (Fig. 5a), involved in ion transport. A single strain contains up to 33 virulence factors (YNHE24), and the minimum number of virulence factors carried by CTX-ESBL-E. coli is 1. We noted differences in virulence factors between chicken farm isolates and clinical isolates, including *fyuA*, *irp2*, *chuA*, *kpsE*, *yfcV*, and *usp*. These virulence genes were significantly and selectively distributed in clinical CTX-ESBL-E. coli isolates (Fig. S1, *P* < 0.0001). Based on an analysis of 24 virulence factors with the highest detection rate in E. coli from different sources, certain differences were observed in the distribution of different virulence factors in the four groups of E. coli. The number of virulence factors of CTX-ESBL-E. coli in each group was analyzed and counted to explore this difference (Fig. 5b). The findings indicate that both clinical isolates from Sichuan Province and those from Yunnan Province contain equivalent virulence factors, which are significantly higher in number than those in chicken farm isolates from Sichuan Province and Yunnan Province. The above results suggest that clinical CTX-ESBL-E. coli could have stronger pathogenicity than chicken farm-derived CTX-ESBL-E. coli.

### Comparison of the relationship between antimicrobial resistance genes, virulence factors, and STs.

**FIG 6:**
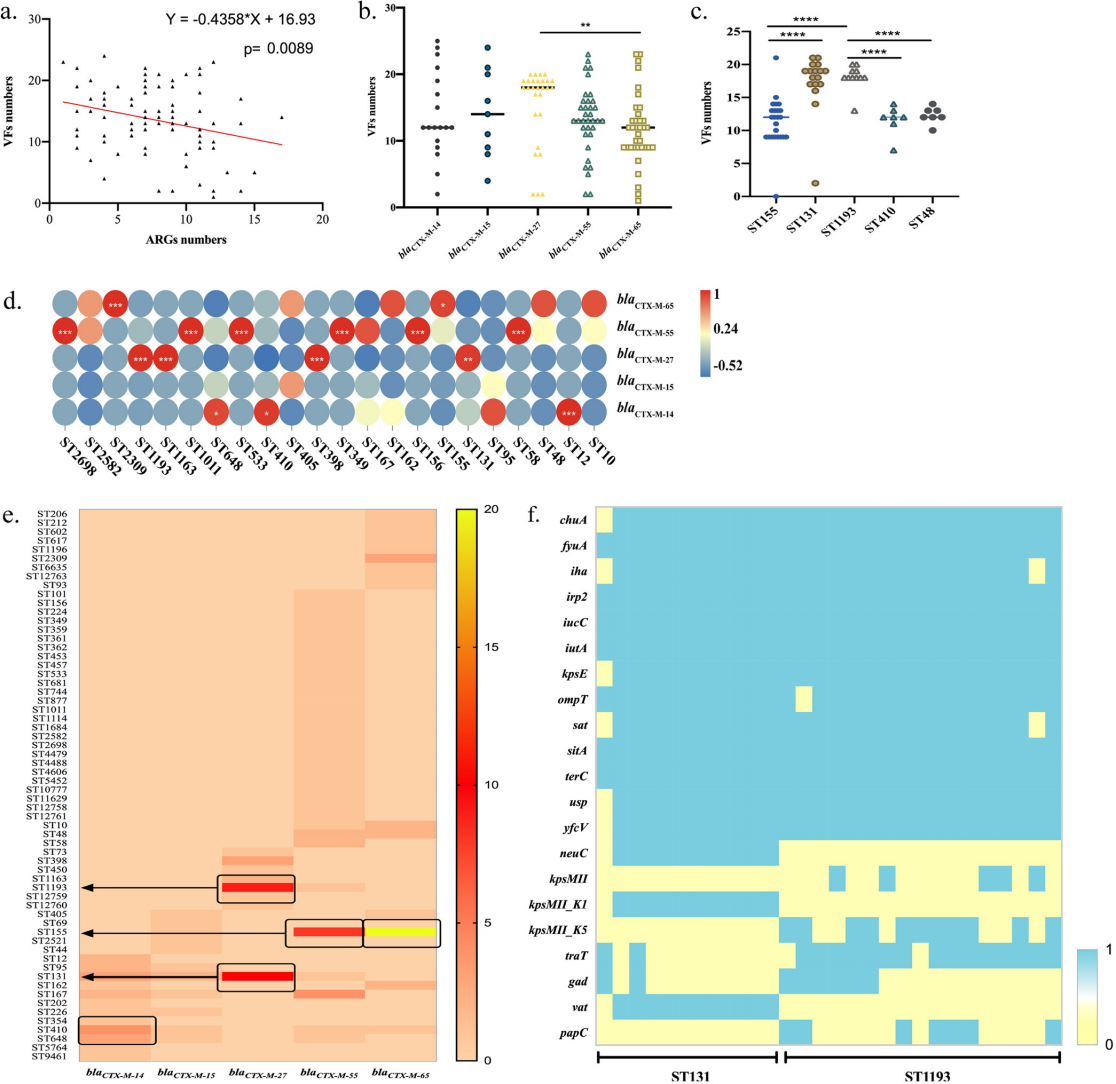
Comparison of the relationships between antimicrobial resistance genes, virulence genes, and STs. (a) Correlation between antibiotic resistance genes and virulence genes. VF, virulence factor. (b) Number of virulence factors in CTX-ESBL-E. coli isolates containing various *bla*_CTX-M_ genes. (c) Number of virulence factors in different STs of E. coli. (d and e) Correlation between *bla*_CTX-M_ genes and STs. *, 0.01 < *P* < 0.05; **, 0.001 < *P* < 0.01; ***, *P* ≤ 0.001; ****, *P* ≤ 0.0001. (f) Virulence factors in ST131 and ST1193 E. coli.

The distribution of different *bla*_CTX-M_ genes in E. coli with different sources was analyzed, and consequently, we found more *bla*_CTX-M_ subtypes in the clinical E. coli isolates than in chicken farm isolates. The clinical E. coli isolates had *bla*_CTX-M-3_, *bla*_CTX-M-14_, *bla*_CTX-M-15_, *bla*_CTX-M-27_, *bla*_CTX-M-55_, *bla*_CTX-M-64_, *bla*_CTX-M-65_, and *bla*_CTX-M-104_, among which *bla*_CTX-M-55_ and *bla*_CTX-M-27_ were dominant. The chicken farm isolates contained *bla*_CTX-M-55_, *bla*_CTX-M-14_, *bla*_CTX-M-27_, and *bla*_CTX-M-65_, among which *bla*_CTX-M-55_ and *bla*_CTX-M-65_ were dominant. Nonlinear regression analysis was used to analyze the relationship between the numbers of drug resistance genes and virulence genes in CTX-ESBL-E. coli. Consequently, we found a negative correlation between antibiotic resistance and virulence genes (Fig. 6a). Subsequently, we counted the number of virulence factors in CTX-ESBL-E. coli containing *bla*_CTX-M-14_, *bla*_CTX-M-15_, *bla*_CTX-M-27_, *bla*_CTX-M-55_, and *bla*_CTX-M-65_ genes. The results showed that E. coli carrying *bla*_CTX-M-27_ had the largest number of average virulence factors, which was significantly higher than that of E. coli carrying *bla*_CTX-M-65_ (Fig. 6b, *P* = 0.0016).

We analyzed the number of virulence factors in ST131, ST155, ST410, ST48, and ST1193 E. coli. According to statistical analyses, the number of virulence factors in different STs varied significantly. Among them, the virulence factors carried by ST131 and ST1193 E. coli were significantly higher in number than those carried by ST410, ST155, and ST48 (Fig. 6c, *P* < 0.0001), further explaining the strong pathogenicity of ST131 and ST1193 as global epidemic high-risk clones. Integrated with *bla*_CTX-M_ subtype analysis, *bla*_CTX-M-27_ was primarily distributed in ST131, ST1193, ST1163, and ST398. Unlike the global epidemic ST131, which was prone to carry *bla*_CTX-M-15_ (32), we found that ST131 is prone to carry *bla*_CTX-M-27_. *bla*_CTX-M-14_ is primarily distributed in ST12, ST410, and ST648 and has a stronger correlation with ST12. Further, *bla*_CTX-M-65_ and *bla*_CTX-M-55_ were distributed in various STs (Fig. 6d and e). ST155 and ST410 E. coli strains were divided into the A group in phylogrouping, whereas ST1193 and ST131 were divided into the B2 group, which is more pathogenic. Therefore, significant attention should be paid to the epidemic characteristics of these high-risk pathogenic E. coli clones.

Later, we analyzed the virulence factors carried by ST131 and ST1193 E. coli (Fig. 6f). The results showed that the ST131 and ST1193 E. coli strains were likely to carry specific virulence factors, including *chuA*, *fyuA*, *iha*, *irp2*, *iucC*, *iutA*, *kpsE*, *OmpT*, *sat*, and *usp* (Fig. 6f). *kpsMII_K1* and *vat* were significantly distributed in ST131 E. coli, whereas *kpsMII_K1* and *traT* were more likely to be distributed in ST1193 E. coli. ST1193 belongs to the ST14 clonal complex (STc14) within E. coli phylogroup B2, which causes human parenteral diseases (33, 34). However, the effects of *kpsMII_K1* and *traT* on the pathogenicity of ST1193 E. coli remain unclear. Studies indicate that the strong adaptability of ST131 E. coli could be related to multiple factors (35). These virulence genes are conducive to improving the versatility and competitiveness of E. coli and the capacity to infect humans. Virulence genes are often located on plasmids; therefore, they are more likely to spread to other E. coli strains with the transferable plasmids, causing changes in the pathogenicity of E. coli. Nonetheless, the precise role of these pathogenic genes remains unexplained.

## DISCUSSION

Excessive use of antibiotics promotes bacterial drug resistance. This work investigated the genomic characteristics of ESBL-E. coli isolated from chicken farms and human medical clinics of Sichuan Province and Yunnan Province, China. The isolation rate of ESBL-E. coli in Sichuan Province was 48% (48/100), whereas that in Yunnan Province was 45.35% (156/344); both were slightly lower than the 58.8% rate in Shanghai (36). We found that the prevalence rate of chicken farm-derived ESBL-E. coli was 34.34% (204/594), which was significantly lower than the 78.6% reported by Liu et al. (37). The above results indicate that the ESBL-E. coli prevalence varied by sources and area from the overall prevalence. Therefore, it is necessary to further analyze the genomic characteristics of ESBL-E. coli in different regions to initiate a reasonable medication strategy. MLST evolution and SNP analyses were performed to analyze the genetic correlation between human and chicken farm CTX-ESBL-E. coli isolates. In MLST analysis, the prevalent STs among 194 strains of CTX-ESBL-E. coli included ST155 and ST131. Among them, the main STs in human CTX-ESBL-E. coli were ST131 and ST1193, which is in line with other reports (38, 39). ST131 and ST1193 are the prevalent STs of E. coli with global high-risk occurrence (34, 40). Of note, ST155 is the primary ST of chicken farm-derived CTX-ESBL-E. coli. The human- and chicken farm-derived CTX-ESBL-E. coli isolates have significantly different STs, as reported by previous studies (41). Additional analyses showed that ST162, ST156, ST10, ST155, ST167, and ST226 are major STs in isolates from humans and chicken farms. This indicates that these STs easily spread between humans and animals. The outcomes of SNP analysis are similar to those of MLST analysis. CTX-ESBL-E. coli isolates from chicken farms and human sources have generally conserved genetic features. However, strains from humans and chicken farms in the same region have a high genetic similarity. The above findings indicate the possibility of cross-transmission of drug-resistant bacteria between poultry and humans in the same region.

Analysis of the resistance genes carried by CTX-ESBL-E. coli revealed that the resistance genes carried by chicken farm-derived CTX-ESBL-E. coli were significantly higher in number than those in their clinical counterparts. This demonstrates that avian E. coli is a vital repository of drug resistance genes. Antibiotic use and antimicrobial-resistant bacterium monitoring in the livestock and poultry breeding industries should be comprehensively managed. These bacteria may be a repository of drug resistance genes and transfer these genes to other pathogens, resulting in clinical treatment failure. Therefore, the high prevalence of antibiotic resistance in chicken farm-derived E. coli presents an urgent demand for the prudent use of antibiotics. Additionally, the isolation rate of *bla*_CTX-M_ in ESBL-E. coli was 82.62%, indicating that the *bla*_CTX-M_ gene is responsible for β-lactam resistance. We analyzed the *bla*_CTX-M_ subtypes carried by CTX-ESBL-E. coli and discovered that the clinical CTX-ESBL-E. coli isolates primarily carried *bla*_CTX-M-14_, *bla*_CTX-M-27_, and *bla*_CTX-M-55_, whereas chicken farm-derived CTX-ESBL-E. coli isolates mainly carried *bl*a_CTX-M-55_ and *bla*_CTX-M-65_, which was consistent with previous reports (42). Notably, the prevalent *bla*_CTX-M_ gene subtypes of CTX-ESBL-E. coli isolates from different sources were different, although *bla*_CTX-M-55_ is a major global epidemic subtype (43). As the conjugative transfer plasmids, IncFII and IncFIb plasmids mediate the horizontal transfer of *bla*_CTX-M_ (44–47). Therefore, the mechanism of resistance gene transfer mediated by plasmids should be further analyzed to manage the spread of drug resistance. In the pathogenicity analysis of CTX-ESBL-E. coli from different sources, we analyzed the phylogroups and virulence factors of 131 CTX-ESBL-E. coli strains. In line with previous studies, the majority of chicken farm-derived E. coli strains belong to the A group, whereas only a few CTX-ESBL-E. coli strains belong to the D group (48, 49). In contrast, above half of human-derived CTX-ESBL-E. coli strains are pathogenic E. coli, primarily belonging to the B2 group. This work suggests that multidrug-resistant E. coli strains of phylogroups A and D have transmission potential between chicken farms and humans, and such subpopulations should be of public health concern. Similar to phylogroup results, clinical CTX-ESBL-E. coli strains appear to carry more virulence factors. Six virulence factors, *fyuA*, *irp2*, *chuA*, *kpsE*, *yfcV*, and *usp*, were significantly distributed in clinical CTX-ESBL-E. coli compared to chicken farm-derived CTX-ESBL-E. coli (see Fig. S1 in the supplemental material, *P* < 0.0001). Several of the above virulence factors are involved in ferric ion uptake and transport, which are typical pathogen characteristics. Therefore, we thought that clinical CTX-ESBL-E. coli could evolve toward higher levels of virulence. Regarding the relationships between drug resistance genes, virulence factors, and STs, E. coli strains carrying *bla*_CTX-M-27_ harbor more virulence factors because *bla*_CTX-M-27_ is extensively distributed in ST131 and ST1193 E. coli. This result is distinct from the ST131 global outbreak, which has *bla*_CTX-M-15_ (50). ST131 and ST1193 are high-risk E. coli clones with more virulence factors than other STs and usually trigger severe infections. Although we did not identify chicken farm-derived ST131 E. coli, ST131 and ST1193 E. coli clones can be transmitted between humans and animals (51). Therefore, prevention and control should focus on ST131 and ST1193 E. coli with *bla*_CTX-M-27_.

The transmission relationship between human- and animal-derived ESBL-E. coli isolates is unclear. Researchers suggest that there is no apparent relationship between human- and animal-derived ESBL-E. coli strains (52). However, there is an indistinguishable association between human- and animal-derived ESBL-E. coli strains, where plasmids are the key vectors driving the horizontal transmission of ESBL genes (53). ESBL-E. coli is an important contaminant in retail chicken meat (54). In this work, a small number of chicken farm-derived E. coli and clinical E. coli isolates were clonally related, suggesting a potential relationship between ESBL-E. coli isolates derived from animals and those from humans. Although human- and avian-origin ESBL-E. coli strains are less clonally related, avian-origin ESBL-E. coli should be comprehensively monitored as an important repository of drug resistance genes (55). In contrast with the phylogenetic relationship between clinical and avian-origin E. coli strains, additional clonally close isolates were isolated within hospitals, indicating that the present spread of clinical resistant bacteria is still dominated by nosocomial transmission. Also, several studies indicate that avian-pathogenic bacteria can be transmitted to humans via the food chain or human-animal contact (43, 56). Although China banned the use of colistin as an animal growth-promoting agent in the livestock farming industry in 2017, antibiotic use for preventive or therapeutic purposes in the broad context of large-scale farming is inevitable. Therefore, strategies for preventing, and measuring the traceability of, drug-resistant bacteria in the farming industry should be implemented. Tracing and exploring the potential animal-human transmission chain provide additional comprehensive insights into understanding resistance in chicken farms. This is critical for developing strategies to minimize antibiotic resistance in chicken farms in different regions.

However, this study has compelling limitations. First, the hospital-derived E. coli strains were isolated from patients and exhibited significant pathogenicity. However, the chicken farm-derived E. coli counterparts were isolated from healthy laying hens and mostly belonged to commensals, causing a large difference between the E. coli strains from the two sources. Therefore, to compensate for possible errors, we also supplemented the genome information of E. coli from the two provinces in the EnteroBase database in the analysis of drug resistance genes and virulence genes. To better understand the transmission relationship of CTX-ESBL-E. coli isolates between poultry and humans, sample sources should be expanded to cover healthy individuals, since chicken farm-derived CTX-ESBL-E. coli isolates are likely to cause asymptomatic infection in patients. Therefore, follow-up studies should expand the scope of sample collection for a more comprehensive analysis.

In conclusion, we used whole-genome sequencing to analyze the epidemic characteristics of clinical and avian-origin CTX-ESBL-E. coli. Moreover, we comprehensively analyzed the epidemic characteristics, transmission patterns, and risk factors of CTX-ESBL-E. coli from the genomic level. Our findings indicate that CTX-ESBL-E. coli has some differences in isolation rates among different regions. Besides, *bla*_CTX-M-55_ is the dominant β-lactam resistance gene of CTX-ESBL-E. coli of both human and poultry origins. Despite the limited link between clinical and chicken farm-derived CTX-ESBL-E. coli isolates, we found multiple identical STs distributed between chicken farm- and human-derived E. coli isolates and even the same clone in part of the chicken farm- and human-derived E. coli isolates. Therefore, significant research attention should be paid to the clinical and avian-origin CTX-ESBL-E. coli strains, as well as to β-lactam antibiotic use in poultry farms. To improve our understanding of the hypervirulent ESBL-E. coli, research should focus on ST131 and ST1193 E. coli carrying *bla*_CTX-M-27_.

## MATERIALS AND METHODS

### Isolation and identification of E. coli and AST.

Chicken anal swabs from 15 chicken farms in Sichuan Province and Yunnan Province were collected between January and September 2021. First, the samples were placed in brain heart infusion (BHI) medium and incubated for preliminary enrichment bacteria. Subsequently, the overnight culture broth was streaked onto eosin-methylene blue (EMB) agar medium and cultured overnight at 37°C. Single colonies with similar morphological characteristics (metal luster colonies) were picked and confirmed as E. coli by colony PCR using 16S rRNA gene primers (F, GAAGCTTGCTTCTTTGCT; R, GAGCCCGGGGATTTCACAT). Meanwhile, the E. coli strains were collected from two grade III hospitals located in Sichuan and Yunnan provinces, respectively.

The ESBL production test and antimicrobial sensitivity test (AST) were performed in line with the CLSI guidelines (Clinical and Laboratory Standards Institute, version M100-Ed31). Ceftazidime, cefotaxime (30 μg), and their compound reagents with clavulanic acid (30 μg/10 μg) were used for the identification of ESBL production in E. coli. The Kirby-Bauer disc diffusion susceptibility test was conducted based on CLSI guidelines, and ceftriaxone (CRO, 30 μg), gentamicin (GEN, 10 μg), levofloxacin (LVX, 5 μg), tetracycline (TET, 30 μg), sulfamethoxazole (SXT, 23.75 μg), fosfomycin (FOS, 200 μg), and florfenicol (FFC, 30 μg) were used as the test antibiotics. E. coli ATCC 25922 was used as a quality control strain. The European Committee on Antimicrobial Susceptibility Testing (EUCAST) breakpoint (v 12.0) was used for interpretation if the CLSI breakpoint was unavailable.

### Analysis of phylogenetic group.

Phylogrouping is a convenient, simple, and rapid technique that differentiates phylogenetic groups (pathogenic, opportunistic pathogenic, or commensal) of E. coli. Based on the work of Clermont et al. (57), three gene segments, *chuA*, *yjaA*, and *TSPE4.C2*, were amplified by PCR and combined to form 4 classes of phylogroups. The judgment basis of the results was as follows: when the three fragments were not successfully amplified or only the *yjaA* gene was successfully amplified, they were classified as group A; when only the *TSPE4.C2* fragment was successfully amplified, it was classified as group B1; when only *chuA* fragments were successfully amplified or both *chuA* and *TSPE4.C2* fragments were successfully amplified, they were classified as group D; when *chuA* and *yjaA* fragments were successfully amplified or all three fragments were successfully amplified, they were classified as group B2. In the four phylogenetic groups, the commensal strains belonged to the A group, the weak-pathogenicity strains belonged to the B1 group, the somewhat pathogenic strains belonged to the D group, and the virulent strains belonged to the B2 group.

### Whole-genome sequencing and bioinformatic analysis.

The genomic DNA (gDNA) of the 131 CTX-ESBL-E. coli strains was extracted using the TIANamp bacterial DNA kit (Tiangen Biotech [Beijing] Co., Ltd., China) following the manufacturer’s recommendation. This gDNA was subjected to short-read whole-genome sequencing (WGS). The WGS results were sequenced using the Illumina HiSeq platform (150-bp paired-end reads with approximately 200-fold average coverage). The clean data were assembled into draft genomes using SPAdes_3.13.0 software. For whole-genome sequence analysis, the phylogenetic tree based on SNPs was constructed using CGE CSI Phylogeny 1.4 with default parameters (58), which were observed using the Interactive Tree Of Life (iTOL v6, https://itol.embl.de/) (58). The genome sequence of E. coli C600 (EC600, GenBank accession no. CP031214.1) was used as a reference. A threshold of 5 SNPs among isolates was considered to show them to be clonally related and likely to have an epidemiological link (59). Sequence types were identified through the ST(s) Find website at EnteroBase (https://enterobase.warwick.ac.uk/species/ecoli/search_strains?query=st_search) (60). A minimal spanning tree was constructed using GrapeTree, version 1.5.061 (https://enterobase.readthedocs.io/en/latest/grapetree/grapetree-about.html) (61), an interactive tree visualization program in EnteroBase. Antimicrobial resistance genes, plasmids, and serotypes were identified using ResFinder 4.1, PlasmidFinder 2.1, and SerotypeFinder 2.0 on the CGE website (62–64).

### Statistical analysis.

Descriptive statistical analyses on percentages with standard deviation and prevalence were performed using functions provided in Excel 2021 (Microsoft Software). All statistical analyses (unpaired *t* test, nonlinear regression analysis) were performed using GraphPad Prism 8 (GraphPad Software). Pearson correlation analysis was used to analyze the relationship between STs and antimicrobial resistance genes with values being shown as follows: *P* > 0.05, no label; 0.01 < *P* < 0.05, labeled *; 0.001 < *P* < 0.01, labeled **; *P* ≤ 0.001, labeled ***; and *P* ≤ 0.0001, labeled ****.

### Data availability.

All 131 E. coli genomes sequenced in this study have been uploaded to the NCBI WGS database associated with BioProject under accession no. PRJNA817453 (see Table S7 in the supplemental material). Also, the draft genomes of 63 E. coli strains (distributed in poultry and human sources from Sichuan and Yunnan provinces, respectively) were downloaded from EnteroBase (Table S7) and included in the phylogenetic analysis.
