# Supplementary figures and images for: Comparative Analysis of Phylogenetic Relationships and Virulence Factor Characteristics between Extended-Spectrum β-Lactamase-Producing Escherichia coli Isolates Derived from Clinical Sites and Chicken Farms
Source: Microbiol Spectr. 2022 Nov 14;10(6):e02557-22. doi: 10.1128/spectrum.02557-22 (PMC9769871; doi:10.1128/spectrum.02557-22)

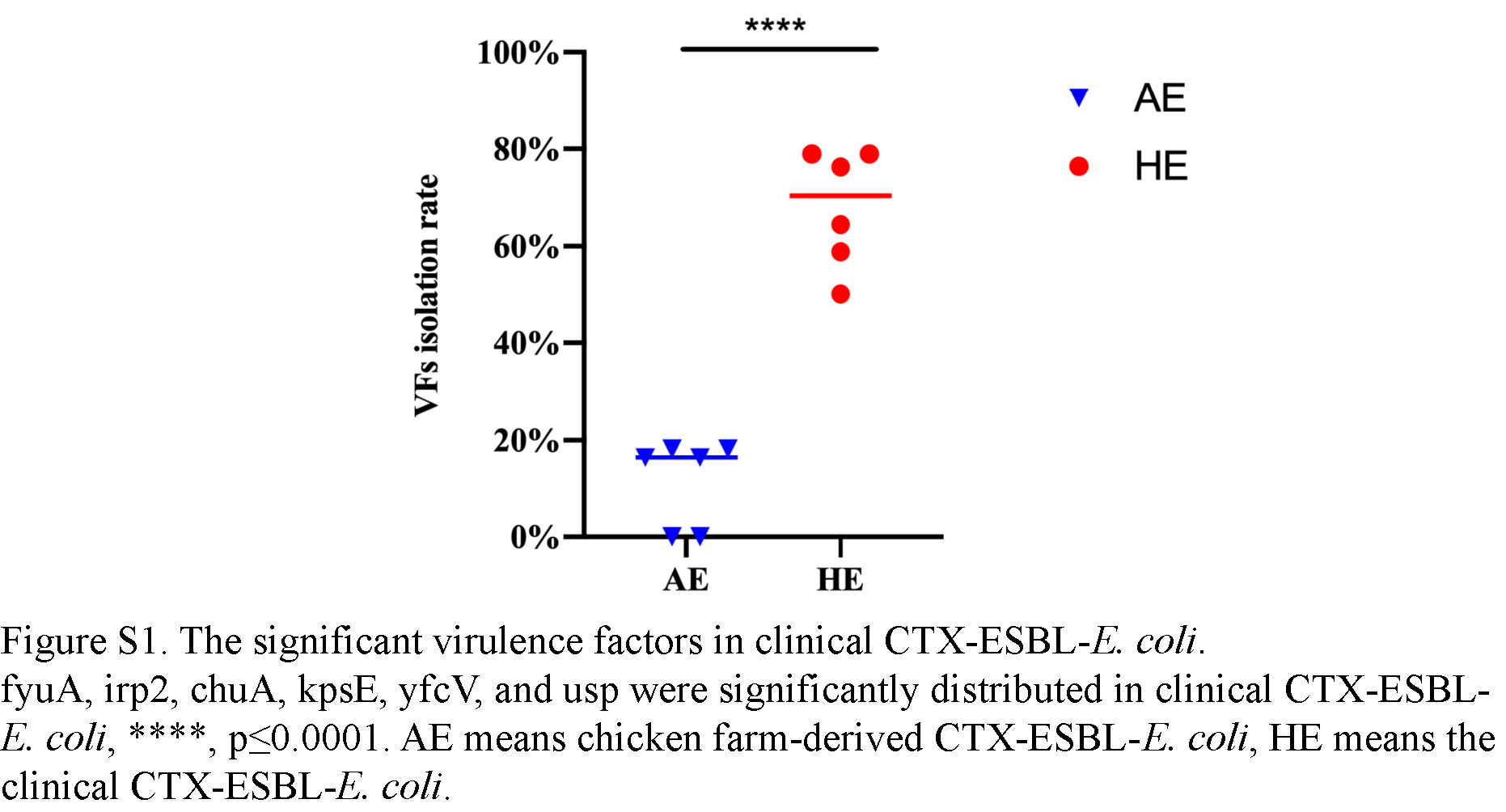

Supplement: Supplemental file 8 — Fig. S1. Download spectrum.02557-22-s0008.tif, TIF file, 0.4 MB [file spectrum.02557-22-s0008.tif]
